# Soft Hydrogels with Double Porosity Modified with RGDS for Tissue Engineering

**DOI:** 10.1002/mabi.202300266

**Published:** 2023-10-31

**Authors:** Bohumila Podhorská, Eva Chylíková‐Krumbholcová, Jana Dvořáková, Miroslav Šlouf, Libor Kobera, Ognen Pop‐Georgievski, Markéta Frejková, Vladimír Proks, Olga Janoušková, Marcela Filipová, Petr Chytil

**Affiliations:** ^1^ Institute of Macromolecular Chemistry of the Czech Academy of Sciences Heyrovského náměstí 2 Prague 6 162 06 Czech Republic

**Keywords:** double porosity hydrogels, hydrogels, mesenchymal stem cells, *N*‐(2‐hydroxypropyl)methacrylamide, scaffolds for tissue engineering

## Abstract

This study develops and characterizes novel biodegradable soft hydrogels with dual porosity based on *N*‐(2‐hydroxypropyl)methacrylamide (HPMA) copolymers cross‐linked by hydrolytically degradable linkers. The structure and properties of the hydrogels are designed as scaffolds for tissue engineering and they are tested in vitro with model mesenchymal stem cells (rMSCs). Detailed morphological characterization confirms dual porosity suitable for cell growth and nutrient transport. The dual porosity of hydrogels slightly improves rMSCs proliferation compared to the hydrogel with uniform pores. In addition, the laminin coating supports the adhesion of rMSCs to the hydrogel surface. However, hydrogels modified by heptapeptide RGDSGGY significantly stimulate cell adhesion and growth. Moreover, the RGDS‐modified hydrogels also affect the topology of proliferating rMSCs, ranging from single‐cell to multicellular clusters. The 3D reconstruction of the hydrogels with cells obtained by laser scanning confocal microscopy (LSCM) confirms cell penetration into the inner structure of the hydrogel and its corresponding microstructure. The prepared biodegradable oligopeptide‐modified hydrogels with dual porosity are suitable candidates for further in vivo evaluation in soft tissue regeneration.

## Graphical abstract

Biodegradable soft hydrogels with dual porosity based on *N*‐(2‐hydroxypropyl)methacrylamide (HPMA) copolymers modified by heptapeptide RGDSGGY significantly stimulate cell adhesion and growth of mesenchymal stem cells also affects the topology of proliferating cells. Thus, these soft hydrogels are suitable candidates for in vivo evaluation in soft tissue regeneration.

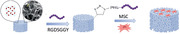

## 1 Introduction

Porous polymer hydrogels with controlled morphology have been widely studied in material research as potential scaffolds for tissue engineering.^[^ ^1^, ^2^ ^]^ Hydrogels applicable for tissue engineering must meet specific criteria, including biocompatibility, biodegradability, pore size and connectivity, suitable elasticity, and mechanical stiffness.^[^ ^3^, ^4^ ^]^ These factors influence the material's suitability for cell adhesion, growth, and differentiation, therefore, developing and designing a suitable hydrogel is essential for successful application.

A wide range of natural and synthetic polymers have been tested and utilized in the synthesis of hydrogels. Natural polymers exhibit biological properties that match the normal tissue microenvironment, promoting desirable cellular responses, biocompatibility, and biodegradability,^[^ ^5^ ^]^ however, they are difficult to reproduce. These polymers are enzymatically degradable but the degradation rate is often difficult to predict.^[^ ^6^ ^]^ Synthetic materials are advantageous due to their chemical and mechanical stability and the possibility of reproducible large‐scale synthesis. Nevertheless, as with natural polymers, these materials do not contain biologically active molecules stimulating cell adhesion and proliferation.^[^ ^6^ ^]^ However, the structure of synthetic matrices can be modified by biomimetic molecules, thus increasing their attraction to growing cells.^[^ ^7^, ^8^, ^9^ ^]^

The morphology of porous soft hydrogels determines successful cell infiltration, as the porosity, pore size, and interconnectivity of the pores in hydrogels significantly impact the properties of matrices. Porosity is significant for cells to acquire oxygen and nutrients and excrete metabolites as waste products.^[^ ^10^ ^]^ Generally, if the pore size is comparable to the cell size and the pores communicate, cells can grow in all three dimensions^[^ ^11^ ^]^ but cell migration is restricted if the pores are too small, leading to the formation of a cellular sheath around the edges of the hydrogel. This can limit the diffusion of nutrients, thereby the formation of necrotic areas within the matrix. Conversely, if the pores are too large, the surface area is reduced, limiting cell adhesion.^[^ ^12^ ^]^ Therefore, using a matrix with a pore size greater than 30 µm will cause the cells to fill the pores, resulting in an insufficient nutrient supply. For this reason, Přádný et al. have designed a new type of hydrogels based on 2‐hydroxyethyl methacrylate (HEMA) copolymers with dual porosity within a single system containing both smaller and larger pores, with the larger pores serving for cell adhesion and the smaller pores allowing for nutrient diffusion.^[^ ^13^, ^14^ ^]^

Water‐soluble copolymers of *N*‐(2‐hydroxypropyl)methacrylamide (HPMA) have been studied for several decades mainly as drug carriers.^[^ ^15^ ^]^ HPMA copolymers are biocompatible, non‐immunogenic, and non‐toxic but their polymer backbone is not biodegradable. Thus, to facilitate their elimination from the body, their molecular weight should be below the renal filtration limit, which is reported to be ≈50 000 g mol^−1^.^[^ ^16^ ^]^ Therefore, higher molecular weight copolymers should contain biodegradable linkages connecting short polymer chains to enable their elimination from the body by kidneys. HPMA has also been utilized in the synthesis of hydrogels.^[^ ^17^ ^]^ The viscoelastic properties of the HPMA‐based hydrogels are similar to those of the developing spinal cord,^[^ ^18^ ^]^ with porous HPMA‐based hydrogels allowing cell migration and new tissue growth composed of glial cells, blood vessels, axons, and dendrites.^[^ ^19^ ^]^ However, they do not allow non‐specific absorption of proteins (fouling) from the medium or blood plasma^[^ ^20^ ^]^ and lead to uncontrolled cell attachment.^[^ ^21^ ^]^ Such properties of HPMA‐based hydrogels enable to control the cell fate and behavior on the surface of the hydrogel matrix. Woorly and co‐workers have extensively studied porous hydrogels based on HPMA copolymers for tissue engineering applications.^[^ ^22^ ^]^ Recently, we studied the morphology of HPMA‐based hydrogels differing in pore size using various microscopic methods, verifying their cytocompatibility, and evaluated the influence of the hydrogel morphology on cell adhesion and proliferation. Nevertheless, to our best knowledge, no paper presenting biodegradable HPMA‐based hydrogels intended for tissue engineering has been published. HPMA‐based hydrogels containing a hydrolytically degradable hydroxylamine linker have been developed in our institute ^[^ ^23^ ^]^ but unfortunately, the degradation rates within tens of hours suitable for drug delivery purposes were too fast for utilization as scaffolds for cell growth.

The surface of HPMA‐based hydrogels does not contain any domains promoting cell adhesion which can be overcome by employing selected signaling domains. In addition, the surface can be functionalized with biomimetic motifs, whereby cells specifically recognize the matrix surface and increase adhesion, engraftment, and/or differentiation. Modification by extracellular matrix (ECM) adhesion proteins such as laminin, fibronectin, or derived peptides (e.g., RGD oligopeptide) can also increase cell adhesion and cytoskeleton organization^[^ ^24^, ^25^ ^]^ allowing covalent and non‐covalent attachment to the matrix surface.^[^ ^4^, ^26^ ^]^ In our previous work, the laminin coating significantly increased the initial cell adhesion on HPMA‐based hydrogels.^[^ ^27^ ^]^ Arginylglycylaspartic acid (RGD) derived from fibronectin is the most widely used biomimetic motif in tissue engineering^[^ ^28^ ^]^ but only a few studies presenting oligopeptide‐modified HPMA‐based hydrogels have been published, e.g., HPMA‐based hydrogels enriched by RGD oligopeptide sequences increased adhesion of mesenchymal stem cells (rMSCs) on the hydrogel surface.^[^ ^22^, ^24^, ^29^, ^30^ ^]^

This paper presents the synthesis and detailed characterization of novel hydrolytically degradable HPMA‐based hydrogels with dual porosity and varying levels of the biomimetic heptapeptide RGDSGGY intended as 3D scaffolds for tissue engineering. Expected benefits of these hydrogels are the improved nutrient supply through additional tiny pores supporting cell adhesion and proliferation, as well as the elimination of the matrix hydrogel from the body.

## 2 Results and Discussion

Biodegradable HPMA‐based hydrogels with dual porosity‐bearing biomimetic oligopeptides RGDS were synthesized and their properties and effect on rMSCs adhesion and proliferation were investigated.

### 2.1 Synthesis and Characterization of Porous HPMA‐Based Hydrogels

**Figure 1:**
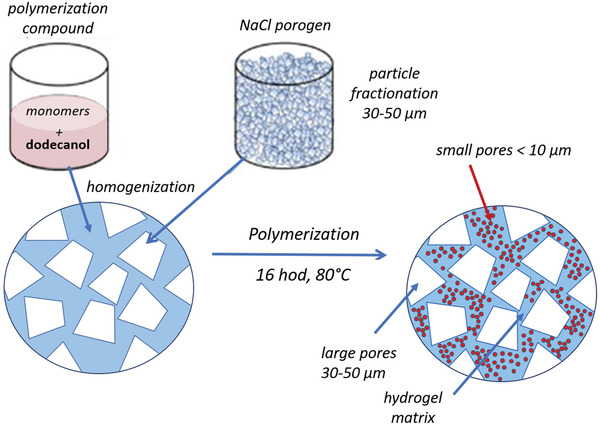
The preparation of the HPMA‐based hydrogel with dual porosity.

The hydrolytically degradable hydrogels were designed based on our findings from our previous study on porous non‐degradable HPMA‐based hydrogels to determine the influence of pore size on cell growth.^[^ ^27^ ^]^ Herein, all hydrogels were prepared by thermally initiated free radical polymerization using a hydrolytically degradable trismethacroylated polycaprolactone (tris(MA‐PCL)) crosslinker. The hydrogel with single porosity (**HS**) was polymerized in the presence of porogen NaCl with the previously optimized porogen size (fraction 30−50 µm)^[^ ^27^ ^]^ and the hydrogels with dual porosity (HD1) contained an additional porogen formed by dispersed 1‐dodecanol.^[^ ^13^ ^]^ The polymerization scheme is shown in **Figure** **1**. The comparison of hydrogel swelling showed a higher swelling capacity of hydrogel **HD1** (93.6 ± 0.3) compared to hydrogel **HS** (83.2 ± 0.5), probably caused by the presence of tiny pores in hydrogel **HD1**. Both polymerization mixtures also contained 1.7% mol. of the positively charged comonomer 2‐(methacryloyloxy)ethyl (trimethyl)ammonium chloride (MOETACl) to improve the initial cell adhesion.^[^ ^31^, ^32^ ^]^ The MOETACl content was optimized to improve the effect of added trimethylammonium cations. However, increasing the range to 4% mol. in hydrogel **HD2** had no impact on rMSCs adhesion compared to hydrogels **HD1** or **HD3** without these groups (see section 2.3), thus, we decided to continue further developing the polymer structure without this co‐monomer. The chemical compositions and schematic structures of the crosslinked copolymers can be found in Supporting Information (Table S1 and Figure S1, Supporting Information).

Hydrogel **HD1** was not suitable for the efficient attachment of biomimetic peptides, thus comonomer *N*‐propargyl methacrylamide (PGMA) was used to synthesize the HPMA‐based hydrogel‐bearing propargyl group (**HP**) (Figure S2, Supporting Information). Their content, determined by ssNMR (10% ± 5% mol. propargyl groups), corresponded with the theoretical amount and was sufficient for further attachment of the RGDS oligopeptide. As expected, the swelling of hydrogel **HP** (93.5 ± 0.5) was comparable to **HD1** since the polymer structure and porogen content were not significantly changed. Samples of hydrogel **HP** had an average value of stiffness, i.e., compressive modulus: 6.8 ± 1.1 kPa. The value is in correspondence with other HPMA‐based hydrogels, e.g. ref.[33] Also, according the same reference, the presence of RGDS oligopeptide did not influenced significantly the stiffness of the hydrogel.

**Figure 2:**
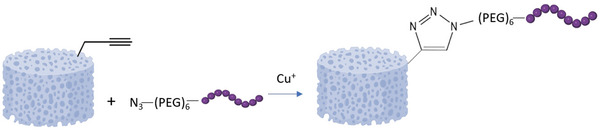
Preparation of hydrogels bearing the RGD motif.

Recently, Golunova et al. showed the benefits of biomimetic peptides on alginates.^[^ ^34^ ^]^ Similarly, we utilized copper‐catalyzed Huisgen 1,3‐dipolar cycloaddition, also called “azide‐alkyne” click chemistry, to attach the RGDS oligopeptide with a short PEG_6_ spacer terminated by the azide group (**Figure** **2**). Two peptide‐modified HPMA‐based hydrogels, **HP1** and **HP2**, were synthesized in one step differing in the oligopeptide content (theoretically 0.3 or 3% mol., respectively) (Figure S3, Supporting Information). The presence of the RGDS oligopeptide was confirmed by comparing selected carbonyl regions of the original and modified hydrogels (Figure S4, Supporting Information), with approximate values of 1% or 2 %mol and the RGDS was subtracted from the remaining propargyl groups (9% ± 5% or 8% ± 5% mol., respectively). Compared to the theoretical amount, the determined peptide content in **HP1** was overestimated, with less peptide bound in **HP2**. However, there was a significant error in the determination in both cases.

Oligopeptide binding to the hydrogel **HP** was demonstrated by XPS spectroscopy. The contributions of the N atoms in the triazole ring and guanidine in the spectrum of N1s appear to overlap considerably with the amide and charged amine contributions of the original **HP** hydrogel, preventing direct identification and accurate quantification of the RGDS oligopeptide. In contrast, the RGDS reaction resulted in two significant differences in the C1s spectra of bioactive hydrogels **HP1** and **HP2** compared to the original unmodified hydrogel **HP** (Table S2 and Figure S5, Supporting Information). The C1s spectra of the modified materials showed increased contributions at 286.3 eV attributed to the PEG_6_ spacer and a new contribution at 289.5 eV indicating the presence of aspartic acid. The absence of azide group contributions in the hydrogels with bound RGDS oligopeptides indicates the chemical binding of the peptide to the hydrogel surface.

Unfortunately, these methods did not reflect the distribution of oligopeptides in the hydrogel, therefore, it is assumed that the peptide distribution is not uniform over the whole hydrogel but in a gradient as the oligopeptide molecules penetrate the hydrogel by diffusion. The penetration was enhanced by adding hydrogel **HP** in acetone to an aqueous solution of the peptide and other reagents, as the hydrogel shrank in acetone and was re‐swollen in water, thereby allowing the oligopeptides to reach a greater depth from the hydrogel surface.

For visualization of the oligopeptide attachment and distribution within the hydrogel, a fluorescent dye 3‐azidocoumarin ^35^was bound via “azide‐alkyne” click chemistry to propargyl groups of hydrogel **HP** instead of the oligopeptide RGDS. Only after “clicking” the 3‐azidocoumarin, did the dye starts to emit the fluorescence far enough from any chromophores in the hydrogels; thus any possible non‐covalent binding can be excluded. Hydrogels **HP‐Dye1** and **HP‐Dye2** represent labeled analogs of the oligopeptide‐bearing hydrogels **HP1** and **HP2**, respectively. For both hydrogels, a gradient distribution of the dye was observed by LSCM, as is documented in Figure S6 and Videos [Link], [Link], [Link], [Link] (Supporting Information) representing the 3D structure of labeled hydrogels.

**Figure 3:**
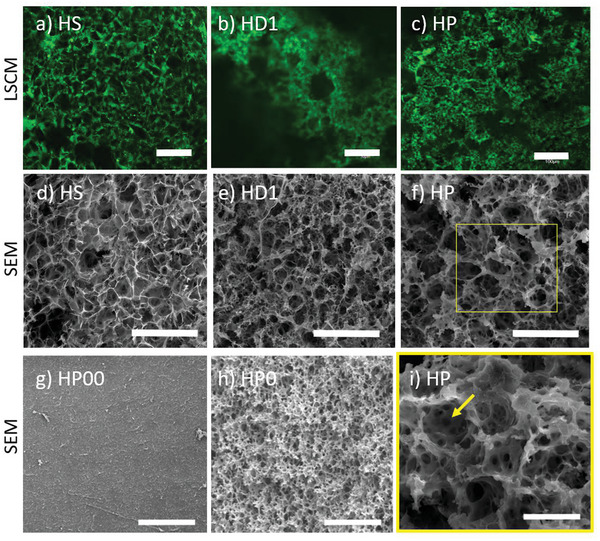
Visualization of the microstructure of HPMA‐based hydrogels swollen in PBS and soaked with fluorescein by LSCM (a–c) and in the dried state by SEM (d–i). Images (a) and (d) show **HS** hydrogel with uniform porosity, images (b) and (e) show **HD1** hydrogel with dual porosity, images (c), (f) and (i) show **HP** hydrogel with dual porosity (the yellow arrow shows microporosity in a large pore). Slide (g) shows hydrogel **HP00** without NaCl porogen and 1‐dodecanol. Image (h) shows hydrogel **HP0** without NaCl but with 1‐dodecanol. Images a‐f) scale bar = 100 µm, g‐i) scale bar = 50 µm.

The hydrogel morphology was investigated in the swollen (LSCM) and the frozen (SEM) states. The LSCM method was applied to samples immersed in the medium at room temperature and is advantageous as the hydrogels are observed in their natural environment, minimizing the risk of artifacts caused by freezing during sample preparation for SEM microscopy. The disadvantages of LSCM include lower resolution, therefore SEM is the preferred method for detailed studies as it provides better contrast and higher resolution. The combination of the two methods provides the most reliable information on the hydrogel microstructure as described in our previous study^[^ ^27^ ^]^ and confirmed the dual porosity of the hydrogels **HD1** and **HP** (**Figure** **3**).

The average pore size of the hydrogel **HS** analyzed by ImageJ from SEM images was 39±5.9 µm, which was in agreement with the range of the porogen fraction used (30−50 µm). The average pore size of hydrogel **HD1** hydrogel was 28±6 µm and **HP** was 25±7 µm. A comparison of the average pore size of the hydrogel **HS** and the hydrogels **HD1** and **HP** shows that the decrease in average pore size is probably due to the dual porosity within one system. The walls of the larger pores contained pores smaller than 5 µm (Figure 3i) with the interconnection of large pores visible as tiny pores in their walls. Both methods confirmed the preparation of a hydrogel with dual porosity. Histograms of pore sizes are summarized in Supporting Information (Figure S7 and Table S3, Supporting Information).

A hydrogel without large pores (HP0) and a non‐porous hydrogel (HP00) were prepared as controls. As expected, hydrogel **HP00** was compact due to the absence of any porogen (Figure 3g), and hydrogel **HP0** contained only small micropores of ∼2 µm due to the presence of 1‐dodecanol during polymerization (Figure 3h). These results are consistent with the formation of larger and smaller pores formed by NaCl and 1‐dodecanol.

### 2.2 Degradation of Porous Hydrogels

**Figure 4:**
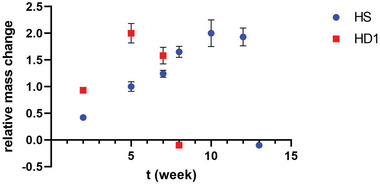
Hydrolytic degradation of porous hydrogel **HS** and hydrogel **HD1** with dual porosity.

As expected, the degradation of hydrogel **HD1** under physiological conditions in PBS at pH 7.4 at 37 °C was very slow (in the order of months, data not shown) due to the slow hydrolysis of oligomeric polyester crosslinkers. Polycaprolactone (PCL) is known to hydrolyze very slowly,^[^ ^36^ ^]^ however, hydrogel **HD1** degraded within eight weeks at 70 °C demonstrating its biodegradability (**Figure** **4**) (The degradation process is shown in Figure S8, Supporting information). The samples initially gained weight due to the increasing water content of the hydrogel structure due to the gradual hydrolysis of the crosslinks. After seven weeks, the sample started losing weight, indicating the early collapse of the hydrogel structure, and then disintegrated to such an extent that it could not be weighed, therefore the relative weight change was recorded as −0.1. It should be noted that the time interval is not absolute because the degradation rate depends on several factors, such as the initial sample size, mass, volume, surface area, diffusion, and the length of chain fragments between bonds.^[^ ^36^, ^37^ ^]^ Moreover, hydrogel degradation can be increased by enzymes, for example, the degradation of PCL by lipases in graft copolymers PCL‐graft‐pHPMA .^[^ ^38^ ^]^ Nevertheless, our system is still a model system, an optimization of the degradation rate should be tailored to the particular application and the target tissue.

The hydrogel **HD1** with dual porosity underwent a faster and shorter swelling (5 weeks) compared to hydrogel **HS** with single porosity (10 weeks), thus more rapid disintegration and degradation, probably due to more tiny pores as discussed in an earlier study on HEMA‐based hydrogels differing in porosity.^[^ ^39^ ^]^ The hydrogels with the smallest pores swelled the most. The time‐dependence curves of the relative mass change showed more pronounced maxima when smaller NaCl particles were used, and the time required for the entire degradation of the hydrogels slightly increased as a consequence of the increase in total pore surface area with increasing size. The hydrolytic reagent had more access to crosslinking between macromolecules, and thus, the time required for degradation decreased. The authors also reported that HEMA‐based hydrogels degraded much faster (on the order of one week) compared to HPMA‐based hydrogels, with an explanation for the different curve shapes and degradation rates based on the significant difference in swelling between HPMA and HEMA‐based hydrogels.

Nevertheless, the authors only reported the disintegration of the macroscopic structure of hydrogels. Polymer fragments consisting of non‐degradable HPMA or HEMA‐based copolymers could still be too big to be easily eliminated by renal filtration. Unfortunately, we did not find any detailed characterization of the molecular weight of such polymer fragments in the literature, so the hydrogel **HD1** was incubated in a borate buffer pH 9.3 at 70 °C to accelerate the hydrolysis of the ester linkages of the crosslinker. The hydrogel disintegrated within a week but the sample was incubated for three weeks to allow hydrolysis of all crosslinks. The molar mass of the polymer fragments was determined by gel permeation chromatography (GPC) after desalting the samples and was *M*_w_ = 820 000 g mol^−1^. To check that this was the final degradation product, HPMA was polymerized under the same reaction conditions used for **HD1** but without the presence of a crosslinker resulting in the linear polymer **HDx**. Therefore, it was assumed that linear polymer chains of the same length would be formed but without the formation of crosslinks. The molar mass of the prepared polymer **HDx** *M*_w_ = 830 000 g mol^−1^ was very similar to that of the degradation fragments of hydrogel **HD1**, thus, confirming the degradation of the polymer network. Nevertheless, due to the very high molecular mass of the polymer fragments, their removal by glomerular filtration cannot be considered as the renal threshold lies between Mw = 50 000–70 000 g mol^−1,^ depending on the structure of HPMA‐based copolymers.^[^ ^16^ ^]^ Therefore, it can be assumed that the slow hepatobiliary route could only remove the water‐soluble polymer fragments from the body.

### 2.3 Cell Cultivation on Porous Hydrogels

The cytocompatibility of the hydrogels was evaluated using rMSCs. Cell viability was above 85% for all hydrogels compared to the control (Figure S9, Supporting Information), suggesting that prepared hydrogels do not induce any cytotoxic effect.

**Figure 5:**
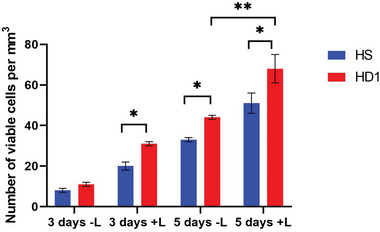
The average number of viable cells growing relative to mm^3^ of porous **HS** hydrogel and dual‐porosity **HD1** hydrogel evaluated after cultivation on days 3 and 5 with (+L) and without (‐L) laminin coating. One‐way ANOVA followed by Tukey's test was used for statistical analysis (**p < 0.01 and *p < 0.05).

Dual‐porosity hydrogel **HD1** and porous hydrogel **HS**, which differed only in porosity, were cultured with rMSCs for 5 days with or without laminin coating. Cells proliferated better but not significantly on the hydrogel **HD1** compared with the hydrogel **HS** (**Figure** **5**). Nevertheless, the improvement was not so considerable compared to the previous results on HEMA‐based hydrogels.^[^ ^13^ ^]^ The initial cell adhesion was probably too low to observe the contribution of tiny pores but a significant favorable effect on the growth of laminin was observed in both hydrogels. Furthermore, laminin improved cell proliferation more significantly in hydrogel **HD1** than in **HS**, suggesting that the higher swelling capacity of hydrogel **HD1** compared to **HS** may affect the adsorption of laminin on the hydrogel surface.

Furthermore, the effect of the content of positively charged quaternary trimethylammonium cations in the structure of hydrogels **HD1** – **HD3** (1.7; 4% or 0% mol. of MOETACl, respectively) on the growth of rMSCs was studied in an independent experiment with laminin coating for 5 days (Figure S10, Supporting Information). A slight increase in cell growth was observed for **HD1** and **HD2** hydrogels containing cations compared to **HD3** hydrogel without a positive charge after 3 days of cultivation (*P < 0.05), which agrees with the previously published study on HEMA‐based hydrogels.^[^ ^31^ ^]^ Nevertheless, after 5‐day cultivation with rMSCs, the differences in cell counts were no longer significant for any of the studied hydrogels, as confirmed by LSCM images (Figure S11, Supporting Information). This experiment, therefore, did not confirm that the presence of a positive charge in the hydrogel structure will increase the adhesion of rMSCs to the HPMA‐based hydrogel.

The HPMA‐based hydrogels were highly hydrophilic and contained no biomimetic moieties, thus, the initial adhesion of rMSCs was minimal compared to the number of seeded cells, ≈90% of the cells adhered outside the hydrogel sample to the culture dish. Nonspecific protein adsorption occurred from the medium by adding fetal bovine serum (FBS),^[^ ^40^ ^]^ thus, assessment of adhesion and cell morphology in the absence of serum can reveal the contribution of various surface ligands to cell‐surface interactions. Without serum, cells remain attached to hydrogels in a spherical shape, as described previously.^[^ ^41^, ^42^ ^]^ In the present study, all experiments were in 10% FBS with most cells remaining spherical and did not expand, reflecting low initial cell adhesion and limited protein adsorption. In general, cells respond best to a moderately hydrophilic surface (contact angle 50−80°) that allows the adhesion of specific proteins in sufficient quantities and, most importantly, in the correct geometric conformation.^[^ ^43^, ^44^ ^]^ This makes specific sequences accessible to the cell integrin receptors to form the desired focal adhesions. The low adhesion of rMSCs was attributed to the highly hydrophilic material. The contact angle of HPMA‐based materials is approximately 40°^[,20]^ which is non‐fouling, i.e., does not allow non‐specific adsorption of proteins and very weak protein adsorption usually detaches protein from the surface even after binding. If proteins are only weakly adsorbed, cells adhere to a small extent but detach prematurely,^[^ ^45^, ^46^ ^]^ thus the prepared **HS** and **HD** hydrogels must be modified specifically with oligopeptides to stimulate controlled cell adhesion.

### 2.4 Cell Cultivation on Porous Hydrogels Bearing RGDS Motif

**Figure 6:**
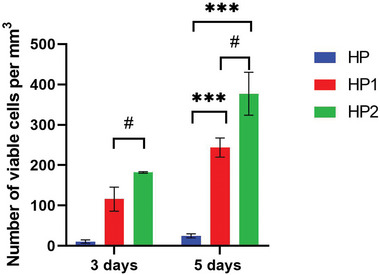
The average number of viable cells growing per mm^3^ of hydrogel **HP**, **HP1**, and **HP2** evaluated after 3 and 5 days of cultivation. One‐way ANOVA followed by Tukey's test was used for statistical analysis (^#^ *p* < 0.05). Dunnett's method was used for individually modified hydrogels versus HP (^***^ *p* < 0.001).

The rMSCs adhered more strongly to **HP1** and **HP2** hydrogels containing the RGDS motif on their surface than to the control **HP** hydrogel without peptide, even without laminin (***p < 0.001, **Figure** **6**). Furthermore, a significant difference in cell proliferation was observed for **HP2** hydrogel compared to **HP1** hydrogel (^#^ *p* < 0.05), with more RGDS bound to the hydrogel providing more accessible specific domains for the rMSCs to interact with integrins.

**Figure 7:**
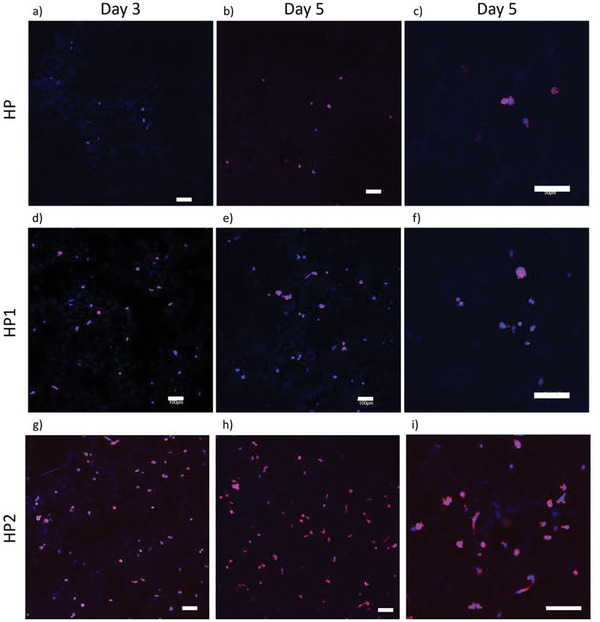
The growth of rMSCs on the hydrogels **HP**, **HP1**, and **HP2** was obtained by LSCM after 3 days (a, d, g) and 5 days (b, e, h) of cultivation. Cells were fixed, and cell nuclei were stained with DAPI (blue) and actin filaments with Rhodamine phalloidin (red). Images (c, f, i) show a close‐up of the cells after 5 days of cultivation. Scale bar = 100 µm.

**Figure 8:**
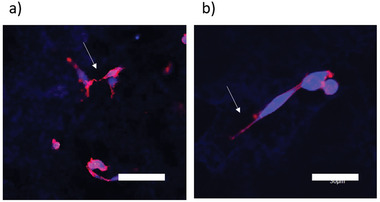
Growth of rMSCs on hydrogel **HP2** was obtained by LSCM after 5 days of culture. Cells were fixed, and cell nuclei were stained with DAPI (blue) and actin fibers with Rhodamine phalloidin (red). Arrows in the images show elongated cells adhered to the hydrogel. Scale bar = 50 µm.

**Figure 9:**
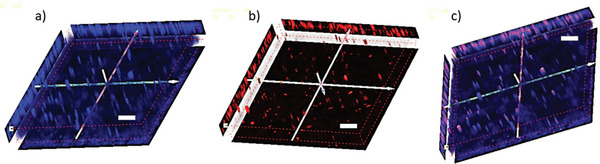
3D visualization of rMSC growth on the surface and inside the hydrogel **HP2** after 5 days of cultivation. Images show cell nuclei in blue (a), actin in red (b), and the overlay from different rotation angles from the axis (c). The 3D structure is composed of 256 files in TIFF format. Scale bar = 100 µm.

Actin filament formation was also assessed in fixed cells with hydrogels **HP1** and **HP2** after 3‐ and 5‐day cultivation, showing sparse elongated actin filaments on day 3 (**Figure** **7**) that significantly increased in number on day 5, and the cells no longer retained only a spherical shape. In hydrogel **HP2**, the effect of elongated cells was more pronounced than in hydrogel **HP1**, with cells having an elongated, even star‐like, flattened shape in places conforming to the hydrogel surface, as illustrated by the arrows in **Figure** **8**. The active cell–cell interaction is documented in Figure 8a, showing that the cells were located not only on the surface of **HP2** but also growing through the hydrogel. The rMSCs were homogeneously distributed on the surface and detected inside the larger pores and on the walls (**Figure** **9**).

The ability of RGDS oligopeptide to expose its active sequences for integrin receptor binding was demonstrated by improved cell adhesion and proliferation on the modified biodegradable hydrogel **HP2**. The RGDS sequence is present in many ECM proteins and serves for cell binding. Studies with substrates coated with RGDS oligopeptides have shown in vitro increases in cell adhesion, migration, proliferation, and differentiation.^[^ ^47^, ^48^, ^49^ ^]^ Therefore, the increased proliferation of rMSCs on RGDS‐modified hydrogels **HP1** and **HP2** can be attributed to the presence of the RGDS sequence.

Compared to native protein laminin, immobilization of oligopeptide with RGDS motif via a PEG_6_ spacer provided a short‐distance interface between the peptide and the hydrogel with greater stability. However, similar behavior was also observed in earlier studies devoted to HEMA‐based hydrogels, where the immobilization of laminin on hydrogel surfaces did not improve the number of adhered cells or their morphology.^[^ ^41^, ^42^ ^]^ It was probably caused by an insufficient spacing or conformation of laminin on the HEMA‐based surface, which are necessary factors for contact with integrins on the cell surface.

## 3 Conclusion

In summary, new hydrolytically degradable soft HPMA‐based hydrogels with dual porosity were developed and characterized. These hydrogels demonstrated higher swelling capacity and hydrolyzed faster than the HPMA‐based hydrogels with uniform porosity, attributed to the presence of more tiny pores and higher swelling capacity. The HPMA‐based hydrogels prevent non‐specific interactions in the cellular environment due to their high hydrophilicity and non‐charged nature. However, cellular experiments with rMSCs demonstrated the cytocompatibility of the obtained hydrogels in vitro thus, their surface modification was optimized to improve cell adhesion and proliferation. The laminin coating improved non‐specific cell adhesion but the positive charge of the quaternary ammonium group in the hydrogel structure did not promote rMSC growth. The most substantial impact on the adhesion and proliferation of rMSCs was observed for the hydrogels with RGDS oligopeptides introduced via azide‐alkyne click reaction. The oligopeptide‐bearing motif derived from fibronectin significantly increased the attractiveness of the scaffold to cells and the increased oligopeptide content and short PEG_6_ spacer exposed the RGDS motif in a favorable conformation, thus increasing ligand availability for cell receptors. Also, the RGDS concentration on the surface of the hydrogel matrix regulated the adhesion, morphology, and metabolic activity of rMSCs and the RGDS‐modified hydrogel allowed rMSCs to grow into the center of the scaffold. Modifying HPMA‐based hydrogels with the oligopeptide RGDS is an effective method for enhancing cell adhesion and proliferation, thus these hydrogels have potential for applications in soft tissue engineering. Further studies should verify the transferability of this method to other stem cells, especially pluripotent stem cells.

## 4 Experimental Section

### Materials for Hydrogel Preparation

*N*‐(2‐hydroxypropyl)methacrylamide (HPMA) was synthesized using a previously described method^[^ ^50^ ^]^ and *N*‐propargyl methacrylamide (PGMA) was prepared according to ^[^ ^51^ ^]^ 2,2`‐Azobisisobutyronitrile (AIBN), 2‐(methacryloyloxy)ethyl (trimethyl)ammonium chloride (MOETACl), 1‐dodecanol, tris(3‐hydroxypropyltriazolylmethyl)amine (THPTA), and trismethacroylated polycaprolactone with an average *M*_n_ = 950 g mol^−1^ (tris(MA‐PCL)) were purchased from Merck‐Sigma‐Aldrich (the Czech Republic). Sodium chloride crystals (250 g) were milled using the laboratory ball mill Fritsch Analysette 3 Spartan overnight. The salt powder was fractionated by sieving overnight utilizing a set of Retsch stainless steel sieves of pore sizes 30−50 µm, respectively (according to standard DIN‐ISO 3310/1). Azidoacetic‐(CH_2_CH_2_O)_6_‐RGDSGGY‐NH_2_ (RGDS‐PEG6‐azide) was prepared by solid‐phase peptide synthesis according to a previously published procedure.^[^ ^52^ ^]^ 3‐Azidocoumarin was prepared according to previously published method.^[^ ^35^ ^]^

### Materials for Evaluation of Cell Growth on Hydrogels

Trypsin, poly‐l‐ornithine, laminin, ethylenediaminetetraacetic acid (EDTA), fluorescein, Triton X‐100, bovine serum albumin (BSA), and dye DAPI were purchased from Merck‐Sigma‐Aldrich. Dulbecco's modified Eagle's medium (DMEM), fetal bovine serum (FBS), water for the cell culture media, AlamarBlue kit reagent, penicillin, streptomycin, Rhodamine‐phalloidin and Hoechst 33 342 were purchased from Thermo Fischer Scientific (the Czech Republic). The mounting medium was obtained from Baria (the Czech Republic).

### Preparation of Hydrogels

All hydrogels were prepared by radical copolymerization of HPMA, comonomers, and a cross‐linking agent tris(MA‐PCL) in the polymerization chamber of a stainless steel tableting apparatus according to a previously described procedure.^[^ ^53^ ^]^ The hydrogels *net*‐poly(HPMA‐*co*‐MOETACl)‐*ν*‐tris(MA‐PCL) (**HD1‐HD3**) with dual porosity were prepared by radical polymerization of HPMA and MOETACl initiated by AIBN using DMSO solvent in the presence of tris(MA‐PCL) crosslinker, NaCl porogen (fraction of 30–50 µm) and 1‐dodecanol. The HPMA, MOETACl, and tris(MA‐PCL) molar ratio was 97.3 : 1.7: 1 for **HD1**, 95: 4: 1 for **HD2,** and 99 : 0: 1 for **HD3**. The molar ratio of HPMA and AIBN was 88: 1. The molar ratio of NaCl, HPMA, and 1‐dodecanol was 20: 1: 0.6. The molar concentration of the monomers in DMSO was 18 m. The polymerization procedure was as follows: 0.5 g of HPMA (3.5 mmol), 35 mg of tris(MA‐PCL) (0.04 mmol), and 13 mg (0.06 mmol) (**HD1**), 32 mg (3.8 mmol) (**HD2**), or 0 mg (0 mmol) (**HD3**) of MOETACl were mixed with 0.2 g of DMSO, then 0.4 g of 1‐dodecanol (2.2 mmol) and 7 mg of AIBN (0.04 mmol) were added. The mixture was dissolved and mixed with 4 g of NaCl porogen (69 mmol) before being homogenized by stirring and polymerized in the polymerization chamber at 80 °C for 16 h. Then, the pellet was placed in a water bath on a shaker at 37 °C in distilled water for 3 days, followed by a concentration series of ethanol (1 day 50%, 3 days 100%, 1 day 50%), 2 days in distilled water and incubated for 2 days in PBS (pH 7.4). The hydrogel *net*‐poly(HPMA‐*co*‐MOETACl)‐*ν*‐tris(MA‐PCL) (**HS**) with single porosity was prepared using the same polymerization conditions as for **HD1** but without 1‐dodecanol. Only the solvent volume was increased to substitute 1‐dodecanol. After cooling, the hydrogel **HS** was placed on a shaker and maintained at 37 °C. Distilled water was changed every day for 10 days for thorough purification.

Polymer **HDx** was prepared under the same reaction conditions as for **HD3** hydrogel but without the presence of a crosslinker: 0.5 g of HPMA (3.5 mmol) was mixed with 0.2 g of DMSO, then, 0.4 g of 1‐dodecanol (2.2 mmol) and 7 mg of AIBN (0.04 mmol) were added, the mixture was dissolved, and 4 g of NaCl porogen (69 mmol; fraction 30–50 µm) was added. The mixture was homogenized by stirring and polymerized in the polymerization chamber at 80 °C for 16 h. First, the product was precipitated into an excess of acetone (200 mL), and the precipitated polymer was isolated by filtration, dissolved in methanol, and precipitated into the same quantity of acetone. The solid product was obtained by decantation followed by washing with acetone and centrifugation. Finally, the product was dried to constant weight. The yield of the reaction was 423 mg.

The hydrogel with dual porosity containing propargyl groups, *net*‐poly(HPMA‐*co*‐PGMA)‐*ν*‐tris(MA‐PCL) (**HP**) was prepared using the same polymerization conditions as for **HD3** with the presence of PGMA. The molar ratio of HPMA, PGMA, and tris(MA‐PCL) was 88.5: 10.5: 1. The molar ratio of HPMA and AIBN was 66: 1. The molar ratio of NaCl, HPMA, and 1‐dodecanol was 22.6: 1: 0.7. The molar concentration of the monomers in DMSO was 18 M. The polymerization procedure was as follows:

0.44 g of HPMA (3.1 mmol), 45 mg of PGMA (0.37 mmol), and 35 mg of tris(MA‐PCL) (0.04 mmol) were mixed with 0.2 g of DMSO, then, 0.4 g of 1‐dodecanol (2.2 mmol) and 8 mg of AIBN (0.05 mmol) were added, the mixture was dissolved and mixed with 4 g of NaCl porogen (69 mmol; fraction 30−50 µm) and homogenized by stirring and polymerized in the polymerization chamber at 80 °C for 16 h. The purification was the same as described earlier for **HD1‐HD3** hydrogels.

Hydrogels **HP0** and **HP00** differing in the presence of porogens were prepared analogously to **HP** hydrogel. Their polymerization conditions were the same as for **HP** hydrogel, except that **HP0** did not contain the porogen NaCl, and **HP00** did not contain NaCl and 1‐dodecanol.

### Attachment of Oligopeptide to Hydrogels

Oligopeptide‐modified hydrogels with dual porosity **HP1** and **HP2** were prepared by a one‐step synthesis using copper‐catalyzed Huisgen 1,3‐dipolar cycloaddition ^[^ ^54^ ^]^ between the azide groups of the RGDS‐PEG_6_‐azide oligopeptide and the propargyl groups of the **HP** hydrogel. In two parallel experiments, ten discs of **HP** hydrogel (corresponding to 100 mg of dry hydrogel containing 0.07 mmol of propargyl groups) were overloaded with a concentration series of acetone (25% for one day, 50% for 2 days, 75% for one day, 100% for one day), then, 2.4 mg of RGDS‐PEG_6_‐azide (0.002 mmol) for **HP1** or 24 mg of RGDS‐PEG_6_‐azide (0.02 mmol) for **HP2** dissolved in 9 ml of distilled water (forming 0.2 mm, or 2 mm solution, respectively) and 0.5 mL of 10 mm sodium ascorbate solution were added to the discs. First, the solution was gently bubbled with nitrogen for 30 min, and then 0.5 mL of 10 mm of the Cu‐THPTA complex solution was added. The reaction was carried out for 24 h at laboratory temperature under an inert nitrogen atmosphere. The discs were washed repeatedly with PBS with 5% w/v EDTA solution until the hydrogels were colorless. Finally, the samples were transferred to PBS. The penetration depth and uniformity of RGDS oligopeptide distribution in hydrogels were studied using a model of 3‐Azidocoumarin staining ^[35]^ The reaction was carried out under the same conditions as the RGDS oligopeptide.

### In Vitro Hydrogel Degradation

The hydrogel hydrolytic degradation dependence was characterized gravimetrically. Hydrolytic degradation of hydrogels **HD1** and **HS** was performed in PBS at physiological pH 7.4 at 37 °C or 70 °C in the case of accelerated hydrolysis and characterized gravimetrically by the relative change in hydrogel mass and expressed as the degree of degradation (*D*) according to Equation (1):

(1) $$D=\left(w_{t}-w_{0}\right)/w_{0}$$

where *w_t_* is the mass of the hydrogel at time *t*, and *w_0_* is the mass of the hydrogel at time *t* = 0. The solution in which the samples were degraded (5 mL of PBS containing 0.5 g L^−1^ sodium azide) was replaced at each weighing.

Hydrogel **HD1** was also hydrolyzed into water‐soluble polymer fragments, and their molecular weight was determined. Then, a disc of the hydrogel was incubated in 4 mL of 0.1 m borate buffer (Na_2_B_4_O_7_·10H_2_O; pH 9.3) in a block heater at 70 °C 30 days. After cooling, the dissolved polymer product was isolated by gel filtration using a PD‐10 column eluted with distilled water followed by freeze‐drying.

### Gel Permeation Chromatography (GPC)

The molecular mass of polymer **HDx** and water‐soluble polymer fragments after hydrolysis of hydrogel **HD1** was analyzed by gel permeation chromatography (GPC) on a Shimadzu (Japan) HPLC system equipped with a TSKGel G4000SW column (7.5 × 300 mm) and a DAWN 8 light scattering detector and an Optilab‐rEX differential refractometer (Wyatt Technology, USA). A mixture consisting of methanol and 0.3 m acetate buffer (pH 6.5) in a ratio of 80: 20% vol. at a flow rate of 0.5 mL min^−1^ was used as the mobile phase. ASTRA VI software was used to calculate the molar masses, using an increment of the refractive index (dn/dc) for HPMA‐based polymers (0.167 mL g^−1^).

### Scanning Electron Microscopy

The detailed morphology of hydrogels was visualized using a high‐resolution SEM microscope (MAIA; Tescan, the Czech Republic). Each hydrogel in triplicate was separately immersed in distilled water (10 mL) for several days. After reaching equilibrium swelling, the samples were cut into sections (10 × 7 × 4 mm) and frozen in a freezer box with liquid nitrogen at – 195 °C. All frozen samples were then freeze‐dried (at a vacuum of 0.1 mbar) for 3 days, cut into thin slices (1.5 mm), and coated with a 4 nm layer of platinum using a LEICA EM SCD 050 sputter coater (Leica, Austria). The sample was observed in an SEM microscope by secondary electron imaging at an accelerating voltage of 3 kV and analyzed using ImageJ software concerning certain limitations according to the previously described procedure.^[^ ^27^ ^]^

### Laser Scanning Confocal Microscopy (LSCM)

An Olympus IX83 confocal microscope with FV10‐ASW software (Olympus, Czech Republic) and a Plan ApoN 40x and 10× objective (1.42 numerical apparatus) was used to study porous hydrogel morphology in vitro and visualize cells adhered to hydrogels. Hydrogel samples were incubated in a fluorescein solution (0.01 %µ) in PBS and the morphology was visualized using LSCM, and the individual planes were scanned using a 10× Plan ApoN objective. ImageJ 1.48 software was used to determine the average pore size of the hydrogels and the pore distribution and to reconstruct the 3D hydrogel structures.

The uniformity of the RGDS oligopeptide distribution throughout the hydrogel was studied using the 3‐Azidocoumarin staining. The slices of the stained hydrogel were excited with a 405 nm laser and scanned in the *Z*‐axis using the 10× Plan ApoN objective of the confocal microscope specified above. The resulting *Z*‐stacks were reconstructed into 3D structures. The slices' flat images on the top, middle, and bottom of the slices were arranged together in the ImageJ 1.48 software.

LSCM was used to visualize live cells and assess cell growth on the materials. After the appropriate cultivation time, the hydrogel samples incubated with cells were transferred to a new well with fresh medium, and 15 min before the scanning, the fluorescent dye Hoechst 33342 (5 µg mL^−1^) was added to stain the cell nucleus. A 405 nm laser was used to excite this fluorochrome, and the emitted light was detected after passing through a 420−500 nm filter. Subsequently, the cells were washed three times with PBS, and the adhered cells were visualized using a confocal microscope.

Cells fixed on the material required a different staining procedure. After the appropriate culture time, the cells on the material were carefully washed three times with PBS and fixed with 4% paraformaldehyde for 15 min. Subsequently, the fixed cells were washed with PBS and permeabilized with 0.1% Triton X‐100 in PBS for 5 min at room temperature. After washing three times with PBS, a 15 min block in 1% BSA was performed, followed by adding specific dyes. Cell nuclei were stained with the addition of 1 µl (1 mg mL^−1^) of the fluorescent dye DAPI and actin filaments with the addition of 2 µL of Rhodamine phalloidin (5 mg mL^−1^) to 0.1% TX‐100 solution in PBS for 20 min at room temperature. The cells were washed with PBS in which they were left or preserved with a mounting medium for longer preservation. Images of cell nuclei were obtained at 100× magnification and analyzed with ImageJ 1.48 using the Analyze particle function.

### Swelling and Porosity of Hydrogels

The equilibrium swelling of the hydrogels in water at laboratory temperature (25 °C) was determined gravimetrically. The water content of the swollen hydrogels was expressed as the degree of swelling (*SD*) according to equation (2):

(2) $$SD=100\times \left(m_{b}-m_{s}\right)/m_{b}$$

where *m_b_* is the mass of the swollen hydrogel at equilibrium and *m_s_* is the mass of dry hydrogels obtained by drying the swollen samples to constant mass in an oven under reduced pressure to 5 mbar at 70 °C. A swelling test was performed in triplicate for each sample, and the average value was calculated.

The volume fraction of pores in swollen HPMA‐based hydrogels Фp was determined using equilibrium swelling of porous hydrogels and swelling of a reference hydrogel matrix prepared without porogen according to a previously described procedure. ^[^ ^27^ ^]^

### Mechanical Properties

The hydrogel **HP** was subjected to compressive testing as described previously.^[^ ^33^ ^]^ The stiffness of the hydrogels was characterized by the compressive modulus of elasticity. The compression test was performed using two cylindrical samples swollen in water using by the Instron 6025R5800 universal testing machine at room temperature. The compressive testing proceeded up to a maximum deformation of 50% of the initial height of the cylindrical sample at a speed of 1 mm min^−1^. Each sample was measured three times in a row, with a pause of about 15 min between measurements to regenerate the sample. The compressive modulus was calculated using linear regression by interpolating points from the initial part of the stress‐strain curve (approximately between 0–5% strain).

### Solid‐State NMR Spectroscopy

Solid‐state NMR (ssNMR) spectra were collected using a 700 MHz Bruker Avance Neo NMR spectrometer (B_0_ = 16.4 T) at Larmor frequencies ν(^13^C) = 176.110 MHz using a double‐resonance 3.2‐mm magic angle spinning (MAS) probe. The ^13^C ssNMR experiments were performed at 20 kHz spinning speed with SPINAL 64 decoupling sequence. ^13^C CP/MAS NMR spectra were recorded with 1.5 ms spin‐lock at 5120 scans and 4 s recycle delay. As external standards, the ^13^C chemical shifts were calibrated using α‐glycine (176.03 ppm; carbonyl signal). The sample was kept and packed into ZrO_2_ rotors under a laboratory atmosphere.

### X‐Ray Photoelectron Spectroscopy

XPS measurements were performed with a K‐Alpha^+^ spectrometer (ThermoFisher Scientific, UK) and the samples were analyzed using a micro‐focused, monochromated Al Kα X‐ray source (400 µm spot size). The kinetic energy of the electrons was measured using a 180° hemispherical energy analyzer operated in the constant analyzer energy mode (CAE) at 200 eV and 50 eV pass energy for the survey and high‐resolution spectra, respectively. The K‐Alpha charge dual‐compensation system was employed using electrons and low‐energy argon ions to prevent localized charge build‐up. Data acquisition and processing were performed using Thermo Advantage software. The XPS spectra were fitted with Voigt profiles. The analyzer transmission function, Scofield sensitivity factors, and effective attenuation lengths (EALs) for photoelectrons were applied for quantification. EALs were calculated using the standard TPP‐2M formalism. All spectra were referenced to the C1s peak of hydrocarbons at a binding energy of 285.0 eV controlled employing photoelectron peaks of PET and metallic Cu, Ag, and Au standards.

### Cells Cultivation

Rat mesenchymal stromal cells (rMSCs), kindly provided by Prof. P. Jendelova, the Institute of Experimental Medicine, the Czech Academy of Sciences, were cultivated in DMEM supplemented with FBS, 100 units of penicillin, and 100 µg ml^−1^ streptomycin in 25‐cm^2^ flasks in a humidified incubator at 37 °C with 5% CO_2_.

### Hydrogel Cytotoxicity

To evaluate the cytotoxicity of hydrogels, rMSCs were seeded in 1 ml of complete medium per 24‐well plate at a concentration of 5×10^5^ cells per well. The next day, hydrogel pieces were added to cell culture inserts (PET membrane, 0.4 µm pore size, Falcon, New York, USA) and incubated for 4 h at 37 °C with 500 µL of the medium. Subsequently, the medium was replaced with fresh medium, and the inserts were transferred to a 24‐well plate with pre‐inoculated cells. After 3 days, the medium and inserts were removed, and fresh medium (300 µ:) containing 10% of the AlamarBlue kit reagent was added to assess cell viability. According to ISO 10993–5, a decrease in cell viability of more than 30% was considered cytotoxic.

### Laminin Coating

Half of the hydrogels studied from each sample were coated with laminin before inoculating cells onto hydrogels. The hydrogel was first cut into 18 mm diameter rounds in its swollen state, and these were subsequently cut into 1/6 sections of maximum 2 mm thickness. They were sterilized by UV irradiation on all sides for 30 min, and then placed in 24‐well flat‐bottomed plates (TTP, the Czech Republic). Overnight, half of the samples were incubated in the solution poly‐l‐ornithine (0.01%) diluted by volume ratio 1:6 with distilled water at 37 °C. The hydrogels were washed with water and incubated in a laminin solution (diluted in DMEM) at a final concentration of 10 µg mL^−1^ laminin at 37 °C for 2 h and 500 µL of DMEM (cell‐free) was added to the wells containing the hydrogels to allow the medium to diffuse into the hydrogels sufficiently.

### Cell Growth on Hydrogel Scaffolds

The rMSCs at a concentration of 1×10^5^ cells mL^−1^ were added to 24‐well flat‐bottom plates containing hydrogel pieces. After culturing the cells on the material for 24 h, the hydrogel pieces were transferred to clean wells of a new plate, and fresh medium was added. At the end of the cultivation, the cells with hydrogels were washed three times with PBS, and cell growth was observed using LSCM. The number of viable cells growing in the hydrogels was assessed using the AlamarBlue cell viability kit containing the active ingredient resazurin. Resazurin was reduced to fluorescently active resorufin by metabolically active cells and its fluorescence is directly proportional to cell viability. At the end of the cultivation period, the hydrogel sample with cells was transferred to a new well of the 24‐well plate, and 300 µL of a new medium with 30 µL of AlamarBlue kit reagent was added. After 4 h incubation, fluorescence intensity (excitation wavelength 560 nm and emission 590 nm) was measured using a Synergy Neo plate reader (Bio‐Tek, the Czech Republic) and converted to cell number using a calibration curve. For the calibration curve, six wells in duplicates were inoculated with cells 24 h in advance, then the cells were washed with PBS and incubated with 0.2 mL trypsin solution (0.05% trypsin and 0.5 mm EDTA in PBS) at 37 °C for 5 min. Trypsin was deactivated by adding 0.2 mL of medium, the solution was aspirated from each well, and the cell pellet was resuspended in 100 µL of PBS. A Büker chamber was used to determine the number of cells, and 0.2% trypan blue was used to assess the number of dead cells and correlate only live cells with the corresponding fluorescence intensity. A calibration curve was constructed as the dependence of the fluorescence of live cells on their number growing on the well plates to determine the number of adhered and growing cells on each piece of the hydrogel according to their fluorescence intensity. A hydrogel sample without cells was used to control and subtract non‐specific backgrounds. The experiment was repeated twice independently in triplicate. Finally, the number of cells was counted and related to the volume of the section (mm^3^). The significant difference in the growth of viable cells on the hydrogel structures was evaluated by one‐way ANOVA.

### Statistical Analysis

The data were analyzed using GraphPad Prism 5.03. Analysis of variance was used to determine statistical significance. The data were assessed by one‐way ANOVA followed by Tukey's method to determine significant differences between the two groups. In addition, Dunnett's method was used for multiple comparisons of samples (individual groups versus control). ^*^ *p* < 0.05; ^**^ *p* < 0.01;^***^ *p* < 0.001; ^#^ *p* < 0.05; ^##^ *p* < 0.01; ^###^ *p* < 0.001.

## Conflict of Interest

The authors declare no conflict of interest.

## Supporting information

Supporting Information

Supplemental Video 1

Supplemental Video 2

Supplemental Video 3

Supplemental Video 4

## Data Availability

The data that support the findings of this study are available from the corresponding author upon reasonable request.
