# Supplementary material for: Soft Hydrogels with Double Porosity Modified with RGDS for Tissue Engineering
Source: Macromol Biosci. 2023 Oct 31;24(3):2300266. doi: 10.1002/mabi.202300266 (PMC13420757; doi:10.1002/mabi.202300266)
Supplement: Supplementary file 1 — Supporting Information [file MABI-24-2300266-s001.pdf]

Macromolecular Bioscience

Supporting Information

for *Macromol. Biosci.*, DOI 10.1002/mabi.202300266

# Soft Hydrogels with Double Porosity Modified with RGDS for Tissue Engineering

*Bohumila Podhorská*, Eva Chylíková-Krumbholcová, Jana Dvořáková, Miroslav Šlouf, Libor Kobera, Ognen Pop-Georgievski, Markéta Frejková, Vladimír Proks, Olga Janoušková, Marcela Filipová and Petr Chytil*

Supporting Information

# Soft Hydrogels with Double Porosity Modified with RGDS for Tissue Engineering

*Bohumila Podhorská*, Eva Chylíková-Krumbholcová, Jana Dvořáková, Miroslav Šlouf, Libor Kobera, Ognen Pop-Georgievski, Markéta Frejková, Vladimír Proks, Olga Janoušková, Marcela Filipová, Petr Chytil*

Institute of Macromolecular Chemistry of the Czech Academy of Sciences; Heyrovského náměstí 2, 162 06 Prague 6, Czech Republic; *corresponding author: podhorska@imc.cas.cz

**Table S1.** Table summarizing the different hydrogel formulations.

| Hydrogel | HS | HD1 | HD2 | HD3 | HP |
|---|---|---|---|---|---|
| **Crosslinker tris(MA- PCL)** | 1%mol. | 1%mol. | 1%mol. | 1%mol. | 1%mol. |
| | | | | | |
| **Co-monomer MOETACL** | 1.7%mol. | 1.7%mol. | 4 %mol. | - | - |
| **Co-monomer PGMA** | - | - | - | - | 10.5%mol. |
| **Porogen NaCl** | 81 %wt. | 78.4 %wt. | 78.4 %wt. | 78.4 %wt. | 77.9 %wt. |
| **1-dodecanol** | - | 7.8 %wt. | 7.8 %wt. | 7.8 %wt. | 8.0 %wt. |

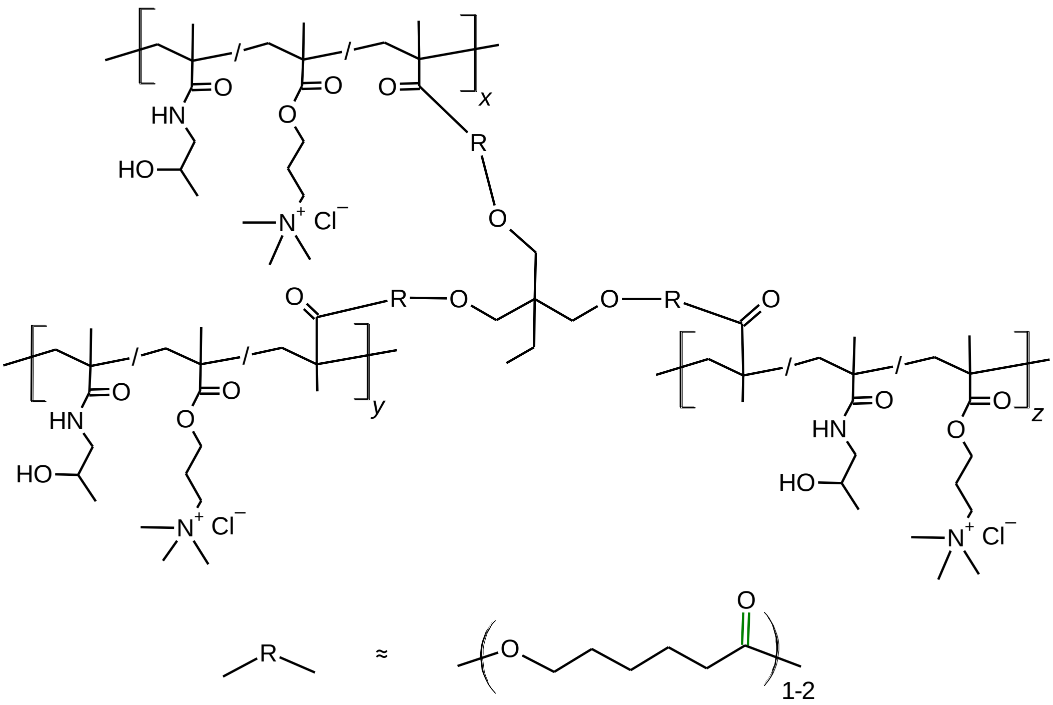

**Figure S1**. Schematic structure of the cross-linked HPMA-based hydrogels **HS** and **HD1 - HD3**, differing in the content of MOETACl.

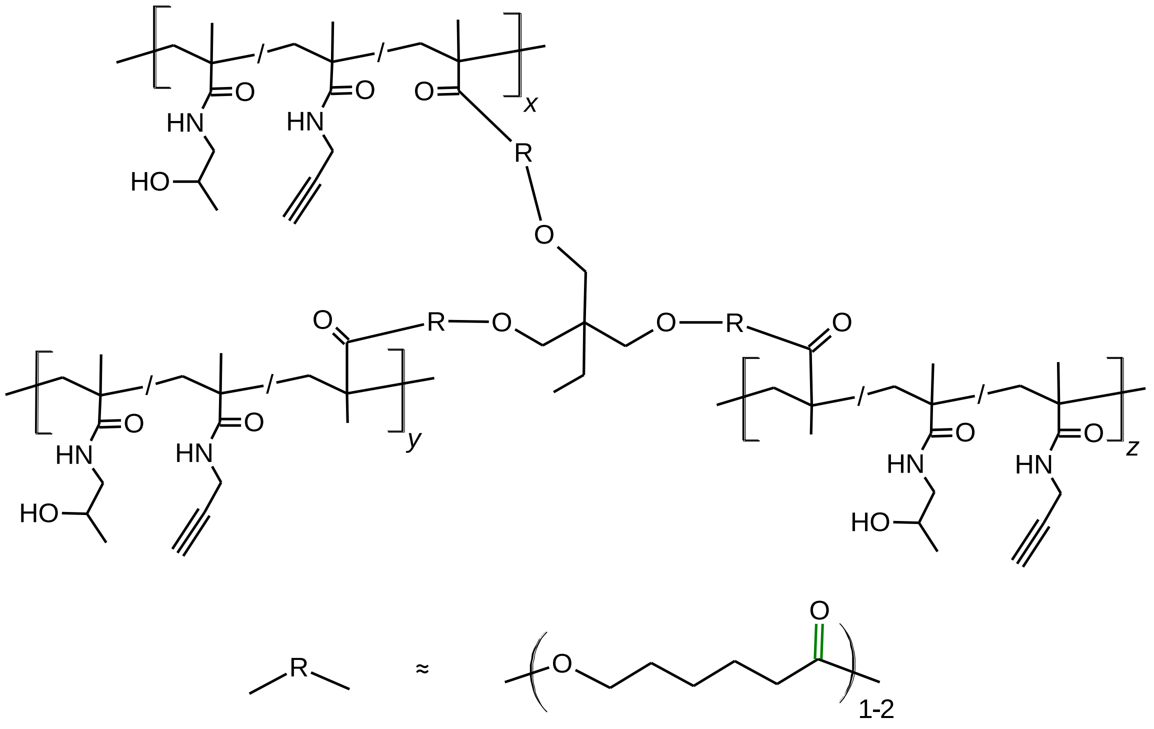


**Figure S2**. Schematic structure of the cross-linked HPMA-based hydrogel **HP**, containing propargyl groups.

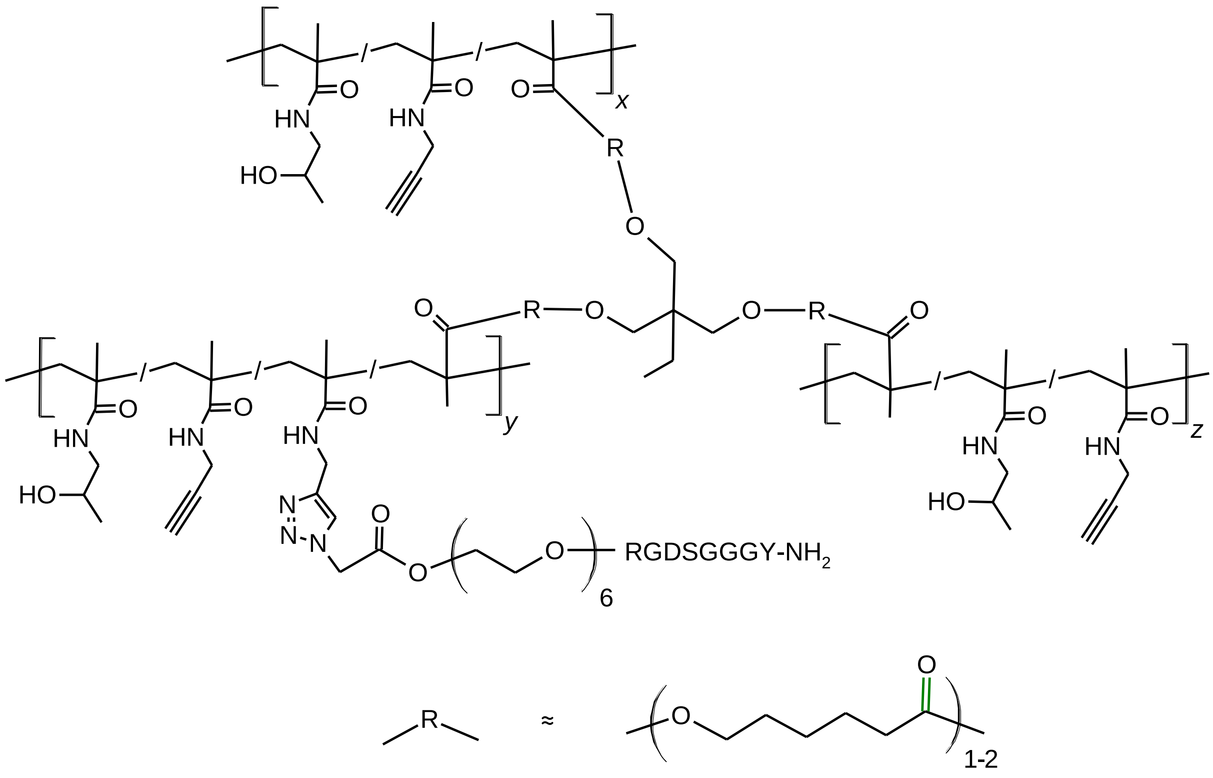


**Figure S3**. Schematic structure of the cross-linked HPMA-based hydrogels **HP1** and **HP2**, containing RGDS peptide bound via the azide-alkyne click reaction.

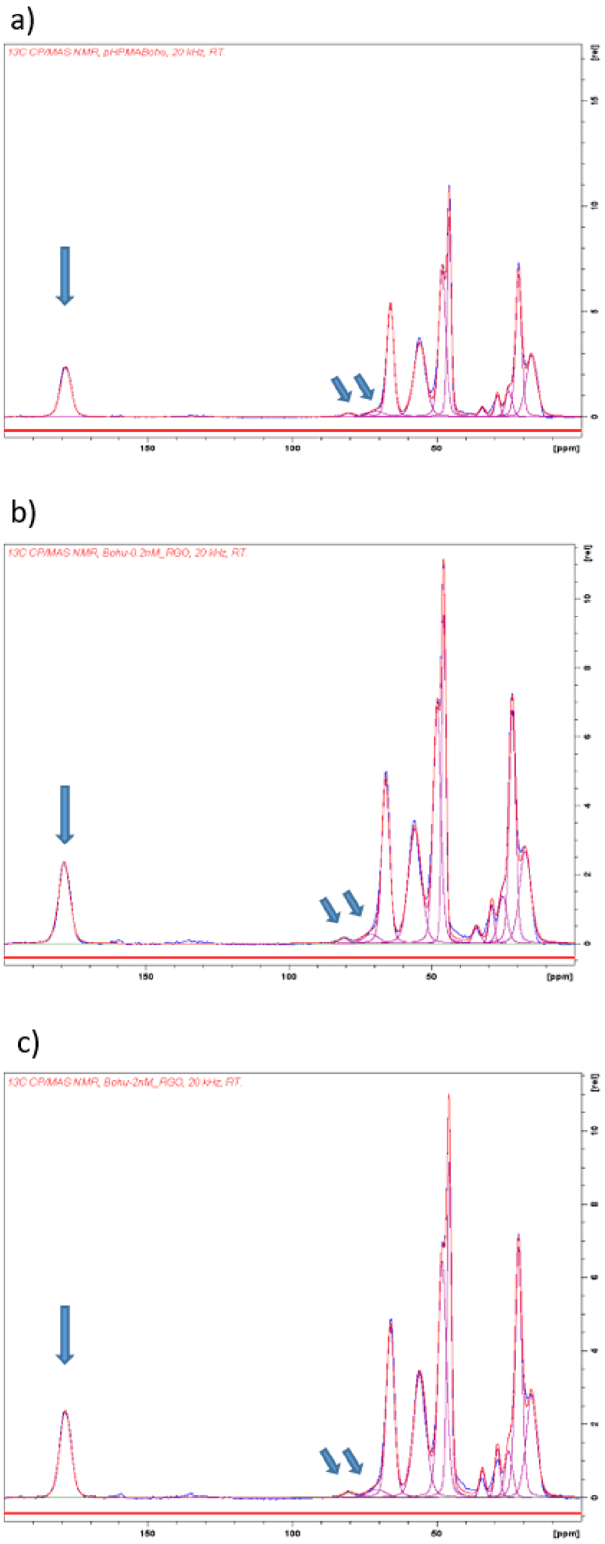


**Figure S4**. Experimental $^{13}C$ CP/MAS NMR spectrum of a) hydrogel **HP** with the corresponding fitting of individual signals. Arrow-marked signals were used for the determination of the mol% of each copolymer. The spectrum of b) hydrogel **HP1** and c) **HP2** with the corresponding fitting of individual signals. Arrow-marked signals were used for the determination of mol% of propargyl groups.

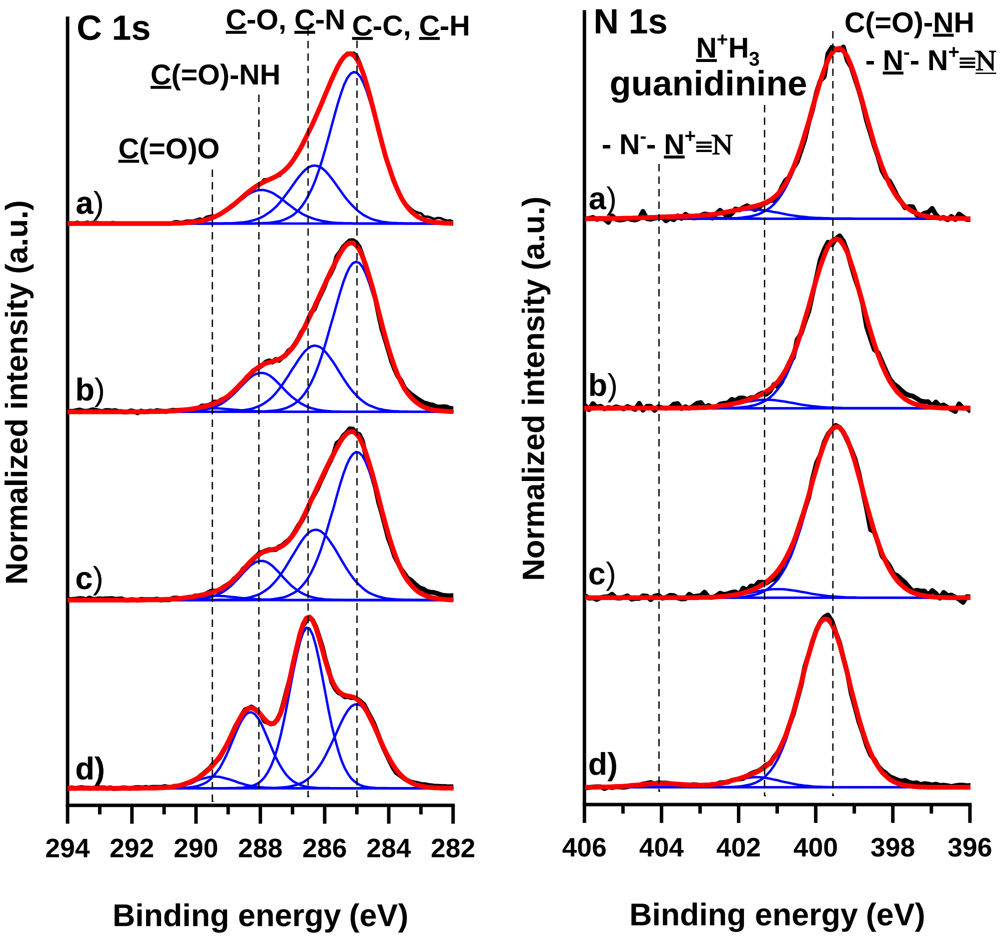


**Figure S5**. High-resolution XPS spectra in the C 1s and N 1s region of a) non-modified hydrogel **HP**, b) hydrogel **HP1** modified with 0.2 mol% RGD, c) hydrogel **HP2** modified with 2.0 mol% RGD and d) pristine RGD peptide. Measured spectra are presented with black lines, while their corresponding fitted envelopes are shown with red lines. The individual contributions of different functional groups are represented with blue lines.

**Table S2.** Atomic % of chemical moieties present on the surface of pristine non-modified **HP**, hydrogel **HP1** modified with 0.2 mol% and hydrogel **HP2** modified with 2.0 mol% RGD, and pure RGD peptide as determined via XPS analysis.

| | C1s C-C | C1s C-O, C-N | C1s C=O-NH | C1s COO | N1s NH-CO | N1s NH+ | N1s azide N+ | O1s |
|---|---|---|---|---|---|---|---|---|
| | Atomic % | | | | | | | |
| **pristine HP** | **45.9±1.5** | **17.6±1.2** | **10.8±0.3** | | **7.8±0.2** | **0.4±0.1** | | **17.6±0.4** |
| **HP1 (RGD 0.2 mol%)** | **44.3±0.5** | **18.8±0.3** | **9.3±0.4** | **1.4±0.2** | **8.4±0.1** | **0.4±0.1** | | **17.5±0.2** |
| **HP2 (RGD 2.0 mol%)** | **42.4±0.8** | **20.1±0.9** | **9.8±0.1** | **1.2±0.1** | **8.1±0.1** | **0.4±0.1** | | **17.9** |
| **Free RGD** | **17.9±1.5** | **24.2±0.7** | **12.3±0.4** | **2.2±0.1** | **15.1±0.5** | **0.9±0.1** | **0.3±0.1** | **27.5±0.6** |

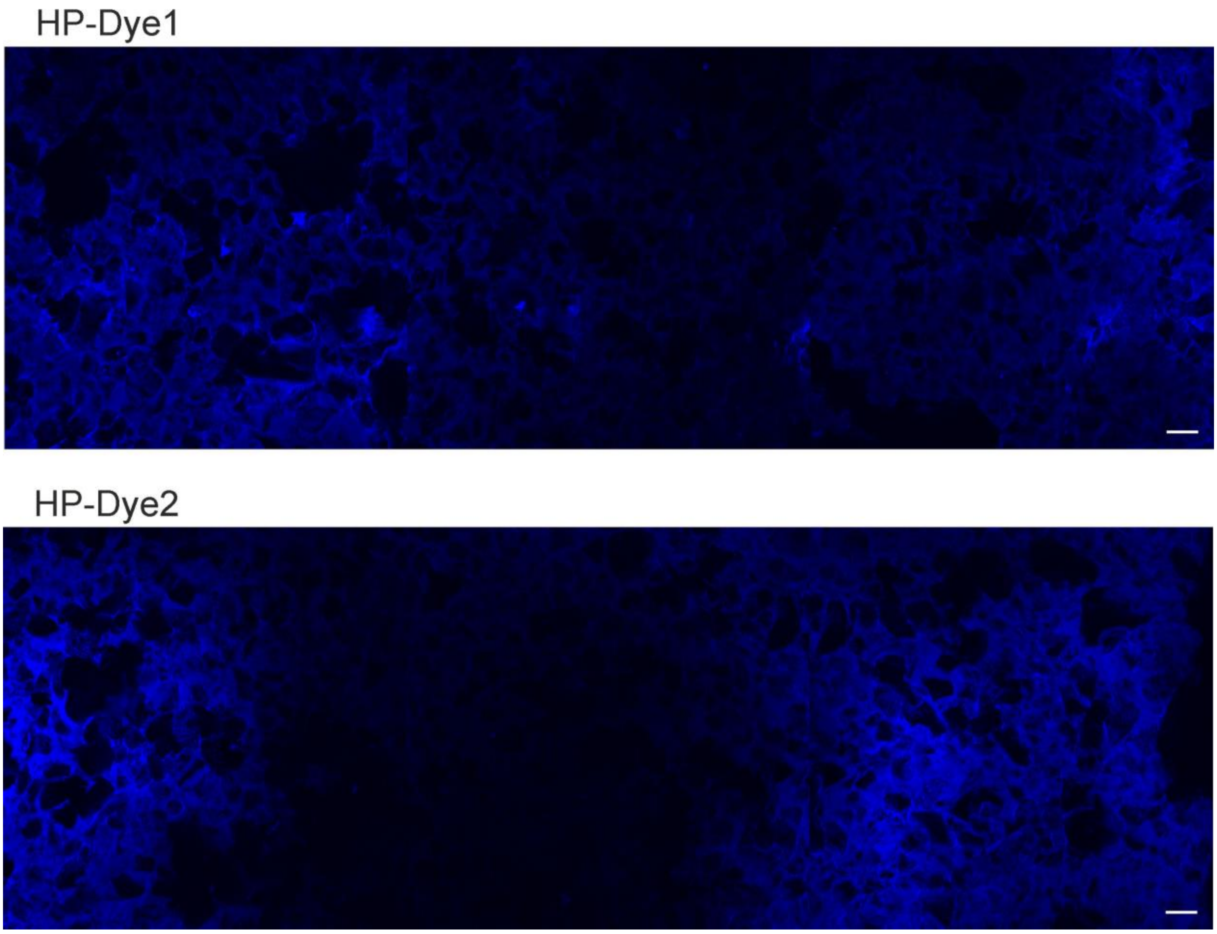


**Figure S6.** Visualization of the distribution of the oligopeptide RGDS to the **HP** hydrogel using the fluorescent dye 3-Azidocoumarin. Hydrogels HP-Dye1 and HP-Dye2 represent labeled analogs of the oligopeptide-bearing hydrogels **HP1** and **HP2**, respectively. Scale bar: 100 µm.

**Table S3.** Characterization of porosity

| **Hydrogel** | **HS** | **HD1** | **HP** | **HP0** | **HP00** |
|---|---|---|---|---|---|
| **Swelling** | 83.2±0.5% | 93.6±0.3% | 93.5±0.5% | 80.6±0.5% | 72.9±0.5% |
| **Porogen NaCl** | 81 %wt. | 78.4%wt. | 77.9 %wt. | - | - |
| **1-dodecanol** | - | 7.8 %wt. | 8.0 %wt. | 7.8 %wt. | - |
| **Min pore size** | 11 µm | 1.4 µm | 2 µm | 0.2 µm | - |
| **Max pore size** | 54 µm | 52 µm | 58 µm | 5.1 µm | - |
| **The average pore size** | 39±5.9 µm | 28±6 µm | 25±7 µm | 2±0.3 µm | - |
| **Median** | 34 | 35 | 36 | - | - |
| **The pore volume fraction ($\Phi p^*$)** | 0.59 | 0.87 | 0.88 | 0.27 | - |

$^*\Phi_P$ is the volume fraction of pores imprinted by porogen calculated from equilibrium swelling

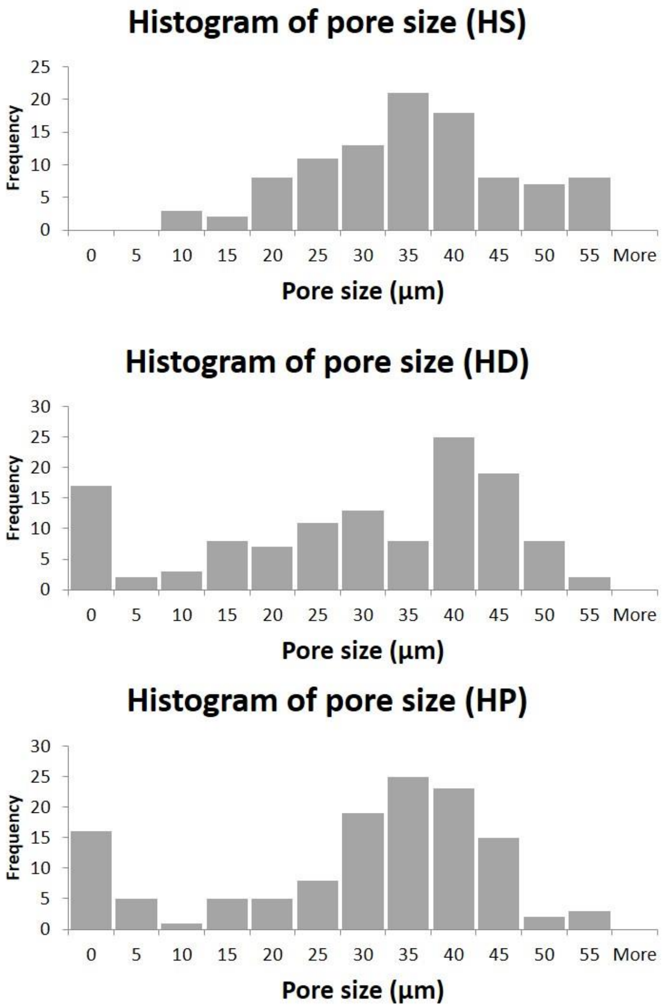


**Figure S7.** Histogram of pore sizes of the hydrogels.

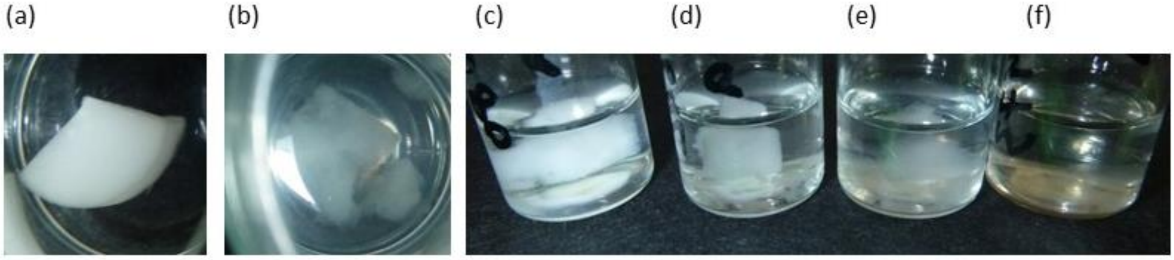


**Figure S8.** Photodocumentation of hydrolytic degradation of HD1 hydrogel at 70°C. a) initial state and beginning of degradation; b) week 2; c) week 3, visible, not disintegrated; d) week 5, visible, disintegrated; e) week 7, slightly visible, disintegrated; f) week 8, completely disintegrated.

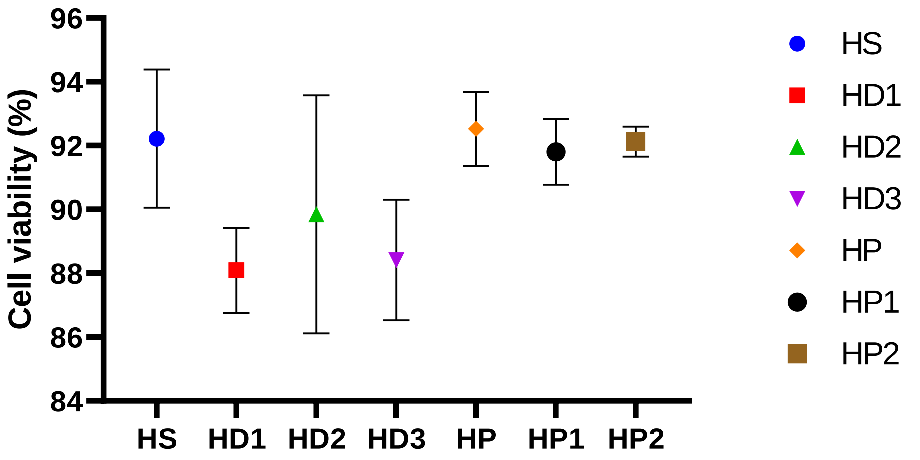


**Figure S9.** The cytotoxicity test. Effect of hydrogels on cell viability determined by resazurin assay after three days.

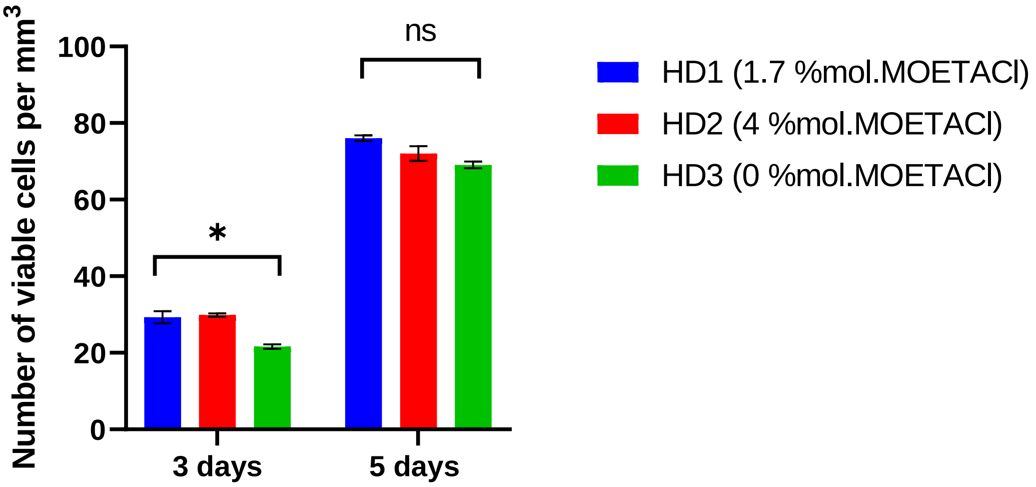


**Figure S10**. The average number of viable cells growing per $mm^3$ of hydrogel HD1 (1.7 %mol. MOETACl), HD2 (4 %mol. MOETACl), and HD3 (0 %mol. MOETACl) evaluated after culturing rMSCs on days 3 and 5. One-way ANOVA followed by Tukey's test was used for statistical analysis. *$P < 0.05$ indicates a significant difference.

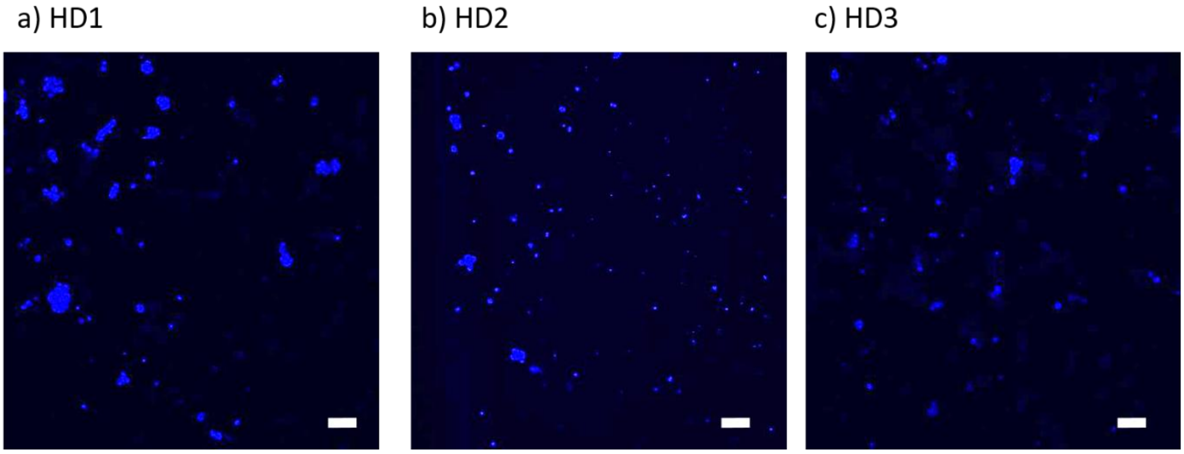


**Figure S11**. Growth of rMSCs on hydrogel of a) HD1 (1.7 %mol. MOETACl), b) HD2 (4 %mol. MOETACl), and c) HD3 (0 %mol. MOETACl) visualized by LSCM on day 5 of cultivation. Cell nuclei are stained with Hoechst 33342 (blue). Scale bar = 100 μm.
